# Cryo-HIM-SIMS on the npSCOPE: Correlative Topographic, Transmitted and SIMS Imaging at Cryogenic Temperatures

**DOI:** 10.1021/acs.analchem.5c06908

**Published:** 2026-04-20

**Authors:** Tatjana Taubitz, Olivier De Castro, Dustin Andersen, Zahraa Berro, Sukriti Hans, Saba Tabean, Moritz Wachsmuth-Melm, Gerhard Hobler, Inge Nelissen, Falk Lucas, Santhana Eswara, Petr Chlanda, Tom Wirtz, Jean-Nicolas Audinot, Antje Biesemeier

**Affiliations:** † Advanced Instrumentation for Nano-Analytics (AINA), Scientific Instrumentation and Process Technology, 87145 Luxembourg Institute of Science and Technology, 4422 Belvaux, Luxembourg; ‡ Department of Structural Biochemistry, Max Planck Institute of Molecular Physiology, 44227 Dortmund, Germany; § Doctoral Program in Systems and Molecular Biomedicine of the DSSE, University of Luxembourg, 4365 Belval Esch sur-Alzette, Luxembourg; ∥ Department of Infectious Diseases-Virology, Medical Faculty, 152528 Heidelberg University, 69120 Heidelberg, Germany; ⊥ BioQuant - Center for Quantitative Analysis of Molecular and Cellular Biosystems, 152528 Heidelberg University, 69120, Heidelberg, Germany; # Institute of Solid-State Electronics, 27259 Technische Universität Wien, 1040 Wien, Austria; ∇ Environmental Intelligence, Vlaamse Instelling voor Technologisch Onderzoek (VITO), 2400 Mol, Belgium; ○ 27219 ScopeM, ETH Zürich, 8093 Zürich, Switzerland

## Abstract

Multimodal helium ion microscopy (HIM) and secondary ion mass spectrometry (SIMS) provide in situ spatial imaging and chemical analysis at nanoscale resolution. Operation at cryogenic temperatures is needed to overcome artifacts related to sample preparation and beam damage during analysis of beam-sensitive samples (including frozen-hydrated specimens). Here, we present a gas field ion source HIM, the “npSCOPE”, that was retrofitted with a liquid nitrogen-cooled copper-shielded instrument chamber and a 5-axis cryo-sample stage. A dedicated liquid nitrogen-cooled ultrahigh vacuum sample transfer system paired with a humidity-controlled cryo-glovebox reducing contamination complements the setup for analysis of frozen-hydrated samples. Thanks to its three detection modalities – secondary electron (SE) imaging, chemical analysis based on an integrated double-focusing magnetic sector SIMS system and scanning transmission ion microscopy (STIM) – this prototype allows for correlative topographic, volumetric and chemical imaging under room temperature (RT) and cryogenic conditions. No significant ice buildup or edge effects were observed when monitoring samples over 36 h at < −139 °C. Cryo-SE imaging confirmed reduced beam-induced modification of beam-sensitive silica-coated gold nanoparticles, and cryo-STIM imaging on a polycrystalline gold foil showed a 34% average improved ion transmission signal intensity as compared to RT. The full cryo-transfer and analysis workflow was successfully tested on cryo-lamellae of plunge-frozen cells prepared by cryo-focused Gallium ion beam milling (cryo-FIB), providing proof-of-principle confirmation for correlative cryo-analysis at the nanoscale. In conclusion, correlative cryo-ion-beam imaging and analysis is a promising solution for nanoscale, multimodal investigations of both close-to-native biological and other beam-sensitive materials.

## Graphical abstract

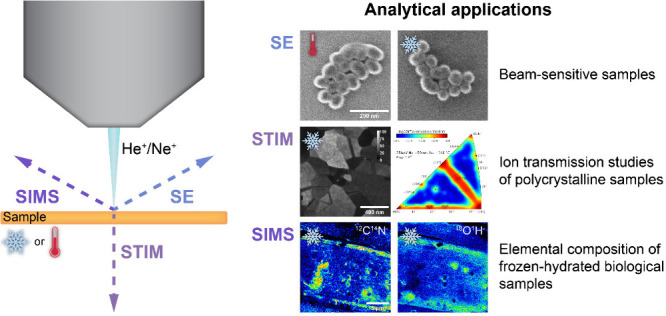

## Introduction

Investigation of samples with charged particle beams at high acceleration voltages offers high-resolution ultrastructural imaging capabilities. At the same time, it presents a significant drawback: the impacting ions or electrons carry significant energy that, even at the limited doses required for imaging, induce ultrastructural alteration or degradation during analysis leading to artifacts in morphology (e.g., viscous flow, melting or sintering effects) and chemical composition (e.g., carbon redeposition or residual hydrocarbon deposition on the sample surface^1^). This is especially critical for soft materials like biological specimens,^1^ organic crystals, polymers, and organic–inorganic hybrid materials, as well as “certain inorganic classes like hydrates and hydroxides”.^2^ Nanomaterials and nanostructures are at even greater risk due to their small size and higher surface-to-volume ratio. While low keV experiments are suitable for some sample types (e.g., 2D graphene^3^), other materials require higher energies, especially if the transmission detection mode is to be used. They can benefit from investigations at cryogenic temperatures, where certain samples are more resistant to beam-induced modification leading to structural damage.

In addition, vitrification methods enable analysis of frozen-hydrated and solvent-rich samples in vacuum. This approach preserves specimens in a near-native state that enables high-resolution ultrastructural analysis with minimal preparation artifacts. Larger tissue pieces need prior manipulation, which can either be done by cryo-focused ion beam (cryo-FIB) thinning^4^ of specific areas of interest for cryo electron tomography (cryo-ET), or by serial milling and block-face imaging of tissues for large volume structural analysis.^5^ Thus, previously unimaginable insights into molecular processes in their subcellular context with down to subnanometer resolution are possible.^6,7^

Chemical composition analyses of biological samples in the frozen-hydrated state are challenging due to the high sensitivity needed for low abundance elemental/molecular signals, e.g., in a physiological intracellular context. Typically, the high exposure times needed for energy-dispersive X-ray spectroscopy (EDX) or electron energy loss spectroscopy (EELS) of low-abundance elements lead to increased beam damage as compared to imaging analysis, ruling out EDX or EELS analyses even in the (cryo-) electron microscope. Focused ion beam (FIB) or combined FIB and scanning electron microscope (FIB-SEM) workstations have the benefit that they allow both imaging and chemical analysis of isotopic signals with higher sensitivity and shorter acquisition times through secondary ion mass spectrometry (SIMS). Very recently, a cryo-NanoSIMS approach was published that allows chemical analysis of frozen-hydrated materials with highly sensitive SIMS at subcellular spatial resolution (O^–^ and Cs^+^ sources with corresponding probe sizes in the order of 40–100 nm).^8^ Direct correlative ultrastructural imaging and analysis based on high spatial resolution secondary electron (SE) imaging and SIMS was recently achieved by incorporating a time-of-flight mass spectrometer onto a cryo-FIB-SEM operating with Ga^+^ beam for the subcellular identification of per-and polyfluoroalkyl substances in bacteria.^9,10^ Another recent work analyzed freeze-dried cryo-lamellae by microscale molecular mass spectrometry after a cryo-ET experiment showing the need for correlative imaging and analysis on near-to-native biological materials.^11^ However, these previously reported chemical imaging methods do not reach close to the theoretical achievable minimum spatial resolution for SIMS imaging (around 10 nm for a Ne^+^ primary beam^12^), which is critical for the correlation of SIMS data with other imaging methods on vitrified samples.

The work presented here reports on the developments toward 20–40 keV ion-based cryo-imaging and cryo-analysis on a prototype instrument platform, the npSCOPE, that was recently developed in our group,^13^ and which is based on a gas field ion source (GFIS). As compared to the FIB platforms above, the GFIS yields minimal probe sizes of <0.5 nm for He^14,15^ and <2 nm for Ne primary beams.^12^ Thus, a spatial resolution of down to <1 nm and an unprecedented depth of field are theoretically possible for He-induced SE imaging, depending on the sample, interaction volume, beam current and acquisition conditions. A scanning transmission ion microscopy (STIM)^16^ detection system can measure variations in the mass–thickness product^17,18^ and the crystalline structure^19–21^ of thin samples (thus making it highly comparable to scanning transmission electron microscopy (STEM)). A double-focusing magnetic sector-based SIMS system collects sputtered secondary ions (SI) from the sample surface and measures their mass/charge ratio, in order to perform chemical analysis and imaging^13^ with a lateral resolution down to <15 nm. All these aspects taken together, the npSCOPE offers a platform for performing multimodal analyses of sample surface morphology, mass–thickness contrast, and chemical composition within the same instrument and with minimal sample handling steps.

While the detailed instrument setup and its multimodal capabilities were already described in a prior Anal. Chem. issue for RT analysis,^13^ this paper focuses on the successful integration of a cryo-stage and cryo-load lock enabling the transfer and investigation of beam-sensitive samples at cryogenic conditions. Benefits for imaging and analysis in these conditions are presented for different material science applications. A highlight is the first-time representation of cryo-SIMS on a frozen-hydrated lamella of eukaryotic cell cultures that paves the way toward near-to-native chemical analysis of life science samples at the highest resolutions and toward the analysis of complex materials with different phases, like hydrogels.

## Experimental Section

### Integration and Technical Assessment of the Cryo-Stage and Cooling System on the npSCOPE

To enable the investigation of frozen-hydrated samples, which must remain below the devitrification temperature (for amorphous ice approximately −137 °C) during sample transfer and imaging, the npSCOPE was equipped with a custom-made high-precision five-axis (X-, Y- and Z-translation, rotation, and tilt) cryogenic sample stage (Steinmeyer, Germany). The cryo-stage and the sample carriers were specifically designed to accommodate all three available imaging modes (SE, STIM, SIMS) without needing to readjust the stage position (See Figure 1 and the graphical abstract). The custom cryo-stage offers true *in situ* correlative microscopy, as all imaging modes are available as long as the sample is placed on a TEM grid and mounted onto the dedicated grid sample carrier (a carrier for stubs is also available).

**Figure 1:**
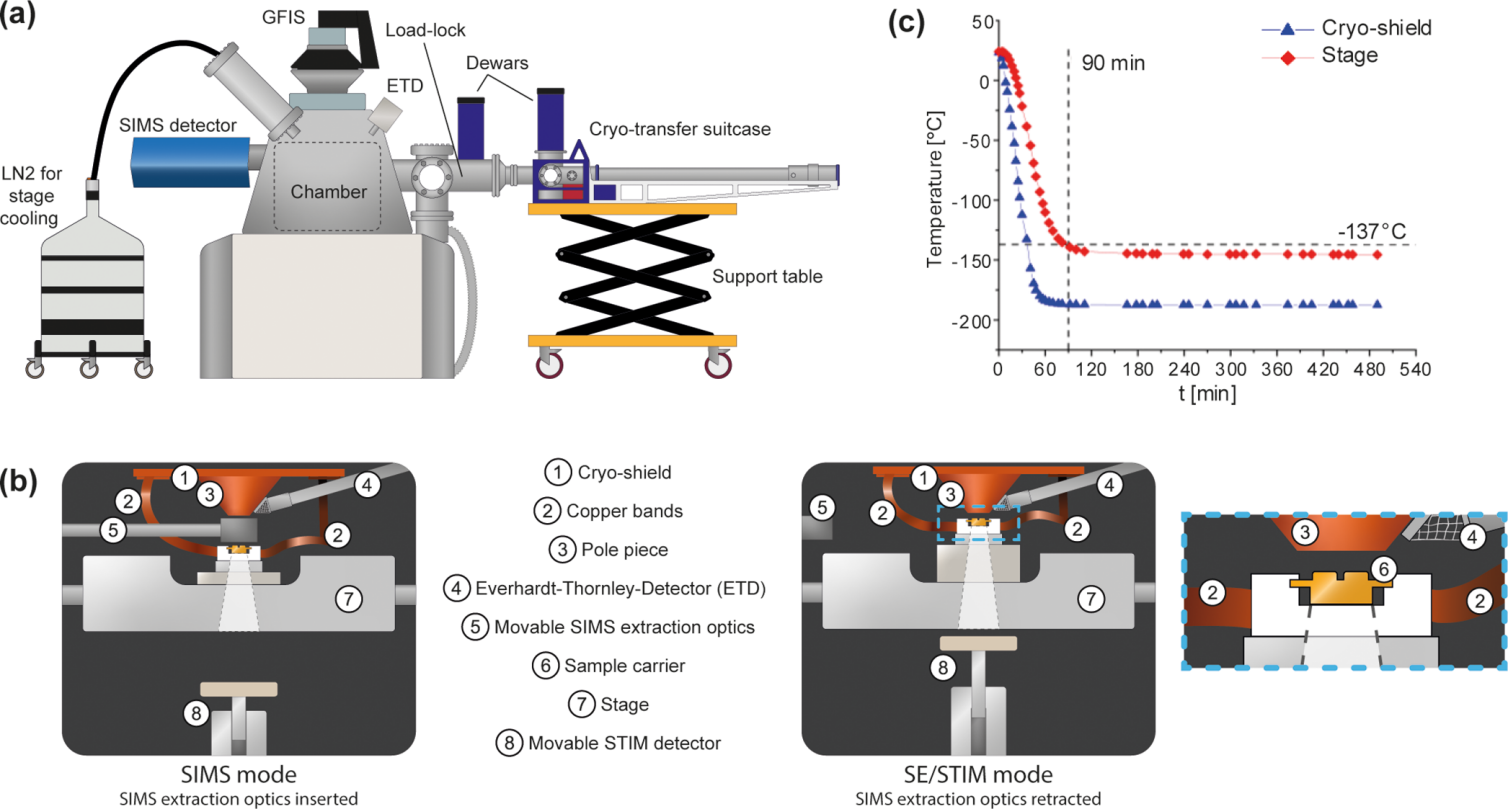
Cryo-modifications on the npSCOPE. (a) Schematics of the outside view with cryo-modifications highlighted. (b) Schematics of the new customized cryo-stage in different modes of operation. Two different views for (left) SIMS and (right) SE or STIM imaging and a zoom into the sample carrier area. (c) Plot depicting temperature drop over time during cooling. Top cryo-shield: *T*_min_ = −188.0 °C, Cryo-Stage: *T*_min_ = −147.5°. Horizontal line at −137 °C denotes the devitrification temperature for amorphous ice.

Cooling of the cryo-stage is achieved via copper bands connected to the stage and cryo-shield, which itself is connected via a coldfinger to an external liquid nitrogen (LN_2_) dewar. An LN_2_ microdosing system (Norhof, Netherlands) maintains the cryogenic temperature for overnight and long-term experiments (Figure 1a,b). Cooling tests demonstrated that the cryo-stage reaches a temperature < −140 °C within less than 90 min after starting to fill the dewar with LN_2_. The stage temperature is stable for another 8 h without additional refills (Figure 1c). In this way, vibrations coming from constant dewar refilling and related LN_2_ “boiling” can be avoided.

To facilitate the transfer of air-sensitive and frozen-hydrated samples into the instrument while reducing the risk of introducing humidity, a cryo-sample transfer system consisting of a dedicated low-humidity glovebox, a cryo-transfer suitcase, and a cryo-load lock was developed together with Ferrovac, Switzerland (now commercially available from Ferrovac, Switzerland^22^). The load lock and transfer suitcase are equipped with high-vacuum systems as well as dedicated cryo-shields and external LN_2_ dewars to avoid any rise in sample temperature or condensation of contaminants onto the sample during transfer (*T* < −180 °C). The glovebox has a liquid nitrogen bath for loading samples into the sample carrier and an LN_2_-cooled high-vacuum loading dock for transfer into the suitcase. For more detailed information about the sample transfer workflow, please refer to the Supporting Information (Supplementary Method 1 and Supplementary Figure S1).

Lack of significant ice build-up while maintaining cryogenic temperatures was validated by investigating a TEM grid over a period of 36h. SE images obtained at different time points did not show any ice crystals. Also, an increase in sample thickness in the grid bar regions, which could be linked to surface ice layer growth, was not observed. Experimental details and analyses are available in Supplementary Method 2 along with the respective results (Supplementary Figure S2, Supplementary Table S1).

### Beam Damage Analysis on Silica-Coated Au Nanoparticles

Silica-coated Au nanoparticles (10 nm Au + 20 nm silica shell, 747564, Sigma-Aldrich, Darmstadt, Germany) were diluted 1:10 in pure ethanol and mixed. A volume of 2 μL was applied onto Formvar/carbon coated Cu finder grids (S162–12, Plano, Wetzlar, Germany) and dried under ambient conditions. This kind of nanoparticle has previously been shown to undergo extensive structural and chemical modification upon helium ion beam exposure at RT.^23^ To evaluate ion beam-induced structural changes at cryogenic temperatures as well, a series of SE images were obtained (30 keV He^+^, 0.7 pA) yielding a comparable ion dose to prior experiments^23^ (here: 1.84 × 10^16^ ions/cm^2^ per image). *Posthoc* data treatment and analyses were performed using *FIJI – ImageJ*. Regions of interest (ROIs) were consistently applied across images to ensure comparative assessment of temperature-related changes. Image alignment was performed using *Scale-Invariant Feature Transform (SIFT)-based registration (linear stack alignment with translation)* to correct for lateral drift. Prior to line profile extraction, a *Gaussian blur* filter was applied to reduce image noise. Intensity line scans (10-pixel width) were extracted from the aligned image stacks to monitor contrast and morphological changes over time. This analysis enabled quantitative comparison of beam-induced effects under RT and cryogenic conditions.

### Ion Channelling in Au (Experiment)

A 50 nm thick freestanding Au membrane (UltrAuFoil, Quantifoil Micro Tools GmbH, Germany) on a 300 mesh Au grid was annealed for 2 h at 700 °C to obtain large grains with isolated twin defects (for a detailed structural analysis of this sample at RT refer to^24^). The sample was cooled to an ultimate temperature of −135 °C using the cryo-stage. While cooling, STIM images were recorded with a 110 μs dwell time, 30 keV beam energy for He^+^, to reach 0.5 pA beam current, yielding a time/temperature image series. The first image in the series is an exception, which was recorded with a 300 μs dwell time, but the measured counts were scaled to be equivalent to 110 μs. The cooling process took approximately 2 h. The detector was placed in the default position of 272 mm beneath the sample for a collection half angle of around 5° (square detector; edge = 5.25°; corner = 7.41°). The entire detector was used, effectively resulting in a bright-field imaging mode.

Due to ion channeling, grains which are well aligned to the ion beam have a higher ion transmission yield and a lower average scattering angle. In a bright-field configuration, this means that the brightest grains correspond to preferred (typically low crystal index) channeling directions, while midgray level grains may either be slightly misaligned or correspond to less preferred (higher-index) channeling directions. The darkest grains are in random orientations to the beam (i.e., no channeling).

Sample drift from cooling was compensated using the *FIJI – ImageJ* feature extraction and image registration plugin *Register Virtual Stack*, set to *Translation*. The image stack was ordered in decreasing temperature, and then ROIs were selected for grains in a variety of orientations as well as for the total image area, and a *Z-axis Profile* was plotted for each ROI.

### Ion Channelling in Au (Simulation)

Simulations of ion scattering were performed to understand the change in transmitted ion intensity over the course of the cooling and imaging. The Monte Carlo binary collision approximation (MC-BCA) software, SRIM,^25^ was used to estimate the damage within the randomly oriented grains (SRIM is not capable of simulating crystalline samples). The random orientation represents the worst-case scenario, since channeling produces gentler interactions. Despite an intermediate dose of 3 × 10^16^ ions·cm^–2^ across all images, the extremely light He^+^ ions are expected to have produced very little displacement damage, with the simulations showing <1 nm of sputtered thickness and approximately 1 × 10^–3^ % vacancies. Furthermore, implantation damage of the type seen in Livengood et al. is not expected to occur – even though similar doses are used – since 75% of the ions are expected to be transmitted through the randomly oriented grains according to the simulation (and even more will be transmitted for channeling orientations).^26^ This means changes in the transmitted ion intensity over the course of the experiment are unlikely to come from damage to the sample during imaging.

A more sophisticated simulation was performed using the IMplant and Sputter sImuLator (IMSIL),^27^ developed at TU Wien. In this simulation, about 1000 different orientations of 50 nm thick single-crystal Au grains were simulated at both RT as well as at −140 °C. The wafer orientations were chosen on a Cartesian grid in a stereographic projection, covering all nonequivalent directions, while the ions’ incidence was perpendicular to the surface. To emulate the effect of the detector collection angle, only ions that left the sample within α = 5° of the surface normal were kept. As a result, channeling maps were obtained, showing the transmission yield as a function of wafer orientation. This technique has previously been used to characterize the orientation dependence of channeling in terms of projected range^28^ and the orientation dependence of the sputtering yield.^29^ In the simulations, the Ziegler-Biersack-Littmark interatomic potential^30^ and a combined Lindhard^31^ and Oen-Robinson^32^ model for electronic stopping, weighted according to a equipartition rule, were used. A correction factor of 1.32 to the Lindhard stopping power was assumed. Target atoms were given random thermal displacements according to the Debye model^33^ with a Debye temperature of −108.15 °C.

### Preparation of Resin-Embedded Bio Specimen

Human HaCaT keratinocyte cell cultures (KeratinoSens, Givaudan Schweiz AG, Kemptthal, Switzerland) were grown according to the OECD Test Guideline for the Testing of Chemicals No. 442D^34^ from 2022 (Dulbecco’s Modified Eagle Medium (DMEM) with GlutaMAX, 9% Fetal Calf Serum (FCS), 0.5 mg/mL Geneticin at 37 °C, 5% CO). Cells were seeded in 6-well plates in growth medium without Geneticin at 1.584.000 cells per well and grown for 24 h before being exposed to 100 μg/mL SiAlTiO_2_ nanoparticles (rutile TiO_2_, >92 wt %, 30 nm in diameter, coated with Silicon and Aluminum, US Research Nanomaterials, Inc.) suspended in DMEM with 1% FCS for 2 h. Next, cells were trypsinzed, collected by centrifugation (300 × *g*, 5 min) and fixed in 2% glutaraldehyde. Then they were dehydrated in a graded series of ethanol washes, stained with 1% OsO_4_ and embedded in EPON resin according to standard procedures. 100 nm thick sections were placed on TEM Cu grids for inspection in correlative SE, STIM and SIMS mode at RT. After a first investigation, the sample stage was cooled down to < −143 °C and the analyses were reproduced with the same acquisition conditions. A detailed description of the correlative workflow for SE, STIM and SIMS at RT and under cryo-conditions as well as a table depicting the standard acquisition conditions at both temperatures can be found in the Supporting Information (Supplementary Method 3, Supplementary Table S2).

Counts versus *m*/*z* ratios were extracted from LIST’s Labview-based VectorView software, and the spectra were replotted using Origin software. Overlay images of different extracted ion maps and imaging modes were created using the *ImageJ Correlia* plugin to overlay the SIMS maps.

### Plunge-Freezing and Cryo-Lamella Preparation by Ga^+^ Cryo-FIB Milling

Glow-discharged gold EM grids (200 mesh, Au, holey SiO_2_, R1.2/20, Quantifoil, Germany) were placed into 35 mm dishes (Corning, USA) coated with polydimethylsiloxane Sylgard 184 (Farnell GmbH, Germany) and disinfected with 70% ethanol for 30 min. The dishes were washed three times with DMEM-F12-GlutaMAX-I supplemented with 10% FCS and 1% penicillin/streptomycin. A549 cells^35^ obtained from American Type Culture Collection (ATCC, USA) were seeded at a density of 2.2 × 10^5^ cells per dish and incubated at 37 °C with 5% CO_2_ for 24 h. Thirty minutes before plunge-freezing, cells were labeled with 3.3 μM Bodipy 493/503 (Life Technologies D3922, USA). Cells were plunge-frozen in liquid ethane at −183 °C using an EM GP2 plunge-freezer (Leica, Germany) with back-side blotting for 4 s. Grids were inserted into FIB-autogrids (Thermo Fisher Scientific, USA) stored in liquid nitrogen until further processing. Cryo-lamella were prepared by cryo-FIB milling using an Aquilos 2 dual-beam cryo-FIB-SEM (Thermo Fisher Scientific, USA) equipped with a cryo-stage at −180 °C. Grids were sputter-coated with Pt (30 mA at 10 Pa) for 10 s before and after deposition of an organometallic platinum protective layer via the Gas Injection System (GIS). Using a 30 keV Ga^+^ beam, cells were semiautomatically milled at a stage angle of 5°. Lamella preparation followed an adapted automated protocol including microexpansion joints.^36,37^ The final two polishing steps were performed manually to obtain lamellae with a nominal thickness of ∼200 nm. After final polishing, the grids were once more sputter coated from the back as described above. Samples were stored and transferred between institutes in nitrogen. After cryo-transfer (Supplementary Method 1), cryo-SIMS analyses were performed with a Ne^+^ beam with a landing energy of 19.5 keV (sample bias of +500 V) or 20.5 keV (sample bias of −500 V) for positive or negative polarity operation, respectively. The ion beam current was set to 12 pA and images were taken with 4 ms dwell time (ion dose of 1.97 × 10^17^ ions/cm^2^ per image). With that relatively high dose, up to 4 consecutive measurements were possible before the lamella ruptured.

## Results and Discussion

### Benefit of Analyses on Beam-Sensitive Samples under Cryogenic Conditions (Silica-Coated Au Nanoparticles)

He^+^-induced beam damage has been previously reported at RT conditions for silica-coated gold nanoparticles.^38^ To also assess beam damage at cryogenic temperatures, structural changes were investigated quantitatively over time under prolonged beam exposure conditions. A series of SE images was acquired at room and cryogenic temperature (see Figure 2a) while using the same ion dose and the same kind of nanoparticles as in the prior research.^38^ Each image obtained corresponds to an ion dose of 1.84 × 10^16^ ions/cm^2^ (1 frame) yielding a cumulative final dose of 1.84 × 10^17^ ions/cm^2^ (10 frames).

**Figure 2:**
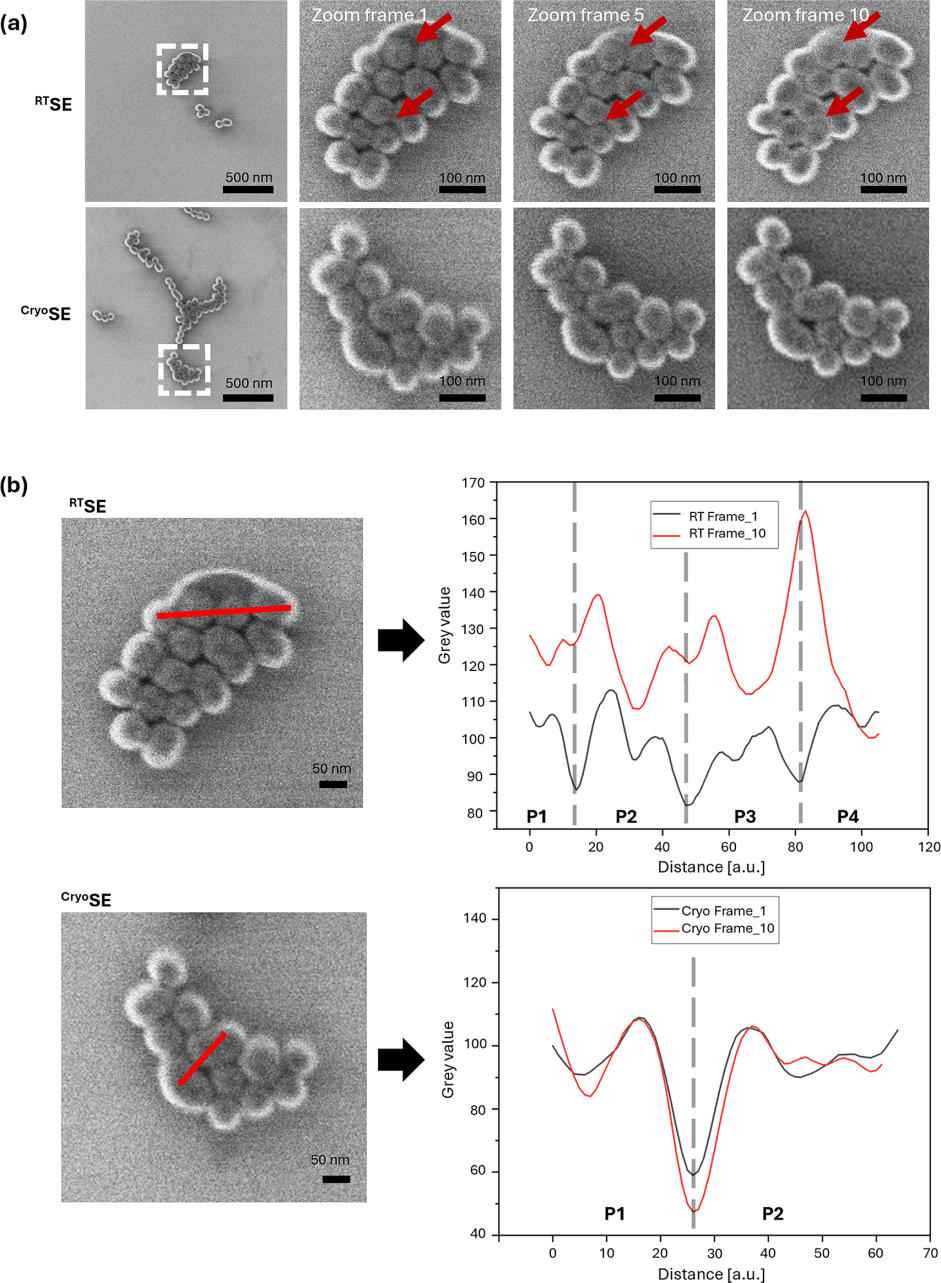
30 keV He^+^ beam-induced damage on 50 nm-sized silica-coated gold nanoparticles after repeated SE (secondary electron) imaging at RT or – 139 °C. (a) Series of 10 consecutive images (total dose of 1.84 × 10^17^ He ions/cm^2^ at RT, upper row) and at another area under cryogenic conditions (lower row). (b) Line scan profiles spanning over two or more particles extracted from the first and last acquisition (frame 1, 10) for each ROI. RT profiles (upper row) reveal beam-induced changes over time. Cryogenic profiles (lower row) show minimal variation, confirming improved particle stability. Vertical dashed guiding lines in the plots indicate particle boundaries along the line profiles. P: particle.

Beam-induced structural alterations, such as ion irradiation-induced viscous flow of the amorphous silica shell and coalescence of neighboring nanoparticles were monitored over time. Gray value line profiles (10-pixel thickness, Figure 2b) were extracted at different positions across the same ROI in consecutive images. Structural alterations were visually identified as reduced gaps between adjacent particles (“interparticle gap”) as well as by less profound “intensity dips” in the line profiles at the valleys in between individual particles (Figure 2b upper row).

A quantitative analysis of the line profiles (Supplementary Results 1 with Supplementary Figures S3–S6 and Supplementary Table S3) revealed that, at RT, particle borders change markedly under repeated beam exposure. The corresponding “intensity dips” in the line profiles decreased on average by ∼87% from frame 1 to frame 10, indicating progressive border smoothing. In some cases, valleys almost disappeared entirely (e.g., P1–P2 in Figure 2b, upper row) or even evolved into continuously rising slopes between adjacent particles (e.g., P3–P4 in Figure 2b, upper row). In parallel, the “interparticle gap” decreased on average by ∼24%, consistent with beam-induced particle coalescence. This suggests that at RT, ion-beam-induced sputtering and material fusion compete, ultimately promoting particle coalescence.

In contrast, under cryogenic conditions, the particle borders remained largely stable. The “intensity dips” changed on average by only ∼7%, with valleys generally remaining unchanged or becoming slightly deeper (e.g., P1–P2 in Figure 2b, lower row). At the same time, the “interparticle gap” increased on average by ∼8%. Therefore, under cryogenic conditions, material fusion is suppressed and no longer counteracts sputtering, resulting in a net material removal under repeated beam exposure.

Nevertheless, whether the observed fusion of particles was an effect of viscous flow, sintering or merely an effect of beam-induced hydrocarbon deposition, the latter being discussed in more detailed in^42,43^ or a combination of these, cannot be fully clarified here. In any case, the reported data shows the expected reduction of beam-induced modification of the sample under cryo-conditions. This improved stability enables longer imaging sessions and detailed analysis of beam-sensitive nanomaterials without compromising morphology.

### Au Foil–Ion Channelling Data

To better understand the impact of the cooling on the STIM imaging mode, measurements of the ion transmission during sample cooling were made for an Au foil – a model system with a variety of grain orientations. Figure 3a shows a representative image from the cooling image series for the Au thin film. The SRIM simulations showed that for a randomly oriented grain, around 70–80% of the ions are expected to be transmitted. However, due to the high degree of scattering for Au, it is expected that >95% of the transmitted counts are lost by scattering outside of the active detector region. For the experimental imaging conditions, an average of 350 incident ions per pixel is expected, resulting in approximately 12–14 counts reaching the detector. This is consistent with the experimentally measured value of ∼ 10 counts per pixel for the random grains at RT.

**Figure 3:**
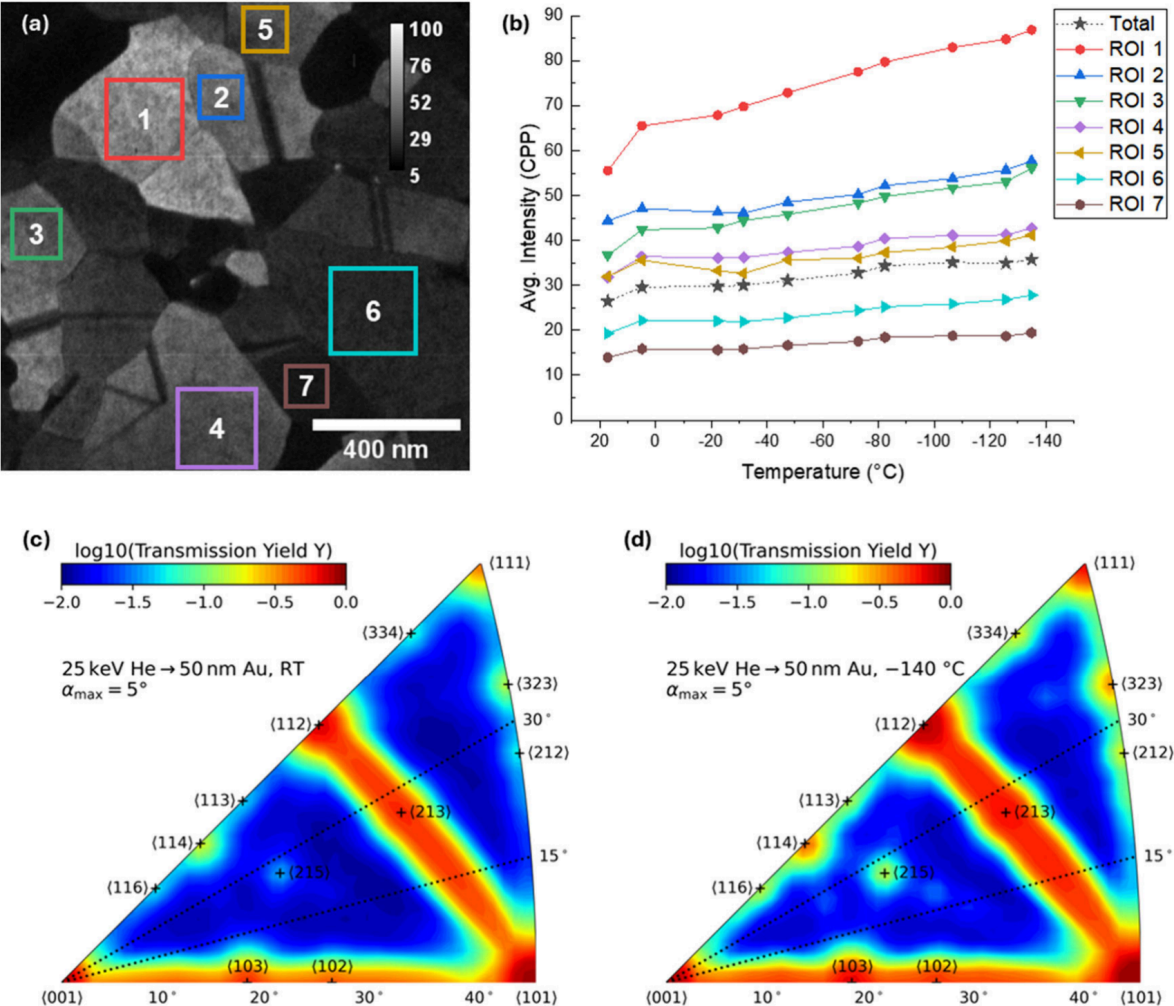
(a) A representative image from the He-based STIM cooling image series with the ROIs indicated. (b) The measured average intensity of each grain within the selected ROI as well as the entire image area (named *Total*) for each temperature. Values are in counts per pixel (CPP). (c-d) Simulations of the ion transmission for 25 keV He^+^ through 50 nm Au across a large range of crystal orientations calculated using IMSIL at (c) room (RT) and at (d) cryogenic temperature. Only ions leaving within α = 5° of the surface normal are collected. On the log scale a value of 0 represents 100% ion transmission, while a value of −2 represents 1%.

Figure 3b shows that the overall intensity of the images went up as the sample was cooled down, with the intensity rising in every grain between RT and cryo. This is consistent with the general expectation of decreased thermal scattering at cryogenic temperatures and is also consistent with the crystalline ion transmission calculations performed with IMSIL, which showed an overall increase of ion transmission at −140 °C as well as a significant enhancement of channeling intensity along higher order crystal directions, e.g., ⟨215⟩ (see Figure 3c,d).

To better compare the results, a least-squares fit was extracted from each of the ROI-temperature curves in Figure 3b and the ratio between RT and cryo-conditions was calculated for the experimental results. This yielded values for the increase in ion transmission ranging from 27% up to 51%, with the total image giving a value of 34%. In a similar manner, the ratio of the simulated ion transmission at RT- and cryo-conditions was calculated for each beam direction shown in Figure 3c,d. There was a large range of values for the increase in simulated transmission with the largest being as high as 250% for a very small number of directions. The orientation of each grain in the experiment is unknown, but the median value (representative of an average grain orientation) of the increase was calculated from simulation results to be 30%. This average simulation value is in excellent agreement with the experimental total image value of 34% (representing an average across many grain orientations).

### Comparison of RT- and Cryo-SE-STIM-SIMS on Resin-Embedded Sections of Biological Cell Cultures

To investigate potential changes in SE, STIM and SIMS acquisition at different temperatures, RT-stable samples – here 100 nm thick cross sections of resin-embedded keratinocytes – were investigated using routine acquisition conditions first at RT (Figure 4a-c) and later under cryogenic conditions (Figure 4d-f). Field of view, instrument settings, acquisition time, and brightness and contrast settings were the same for both temperatures validating the stability of parameters independent of the temperature. Exemplary correlative SE, STIM (Figure 4a) and SIMS data sets (Figure 4b,c) of keratinocytes exposed to 30 nm thick SiAlTiO_2_ nanoparticles are depicted. These resemble samples that are typically investigated on the npSCOPE for nanotoxicological analyses as only FIB-SIMS has the spatial resolution and sensitivity required to identify single nanoparticles in the biological matrix.^13^

**Figure 4:**
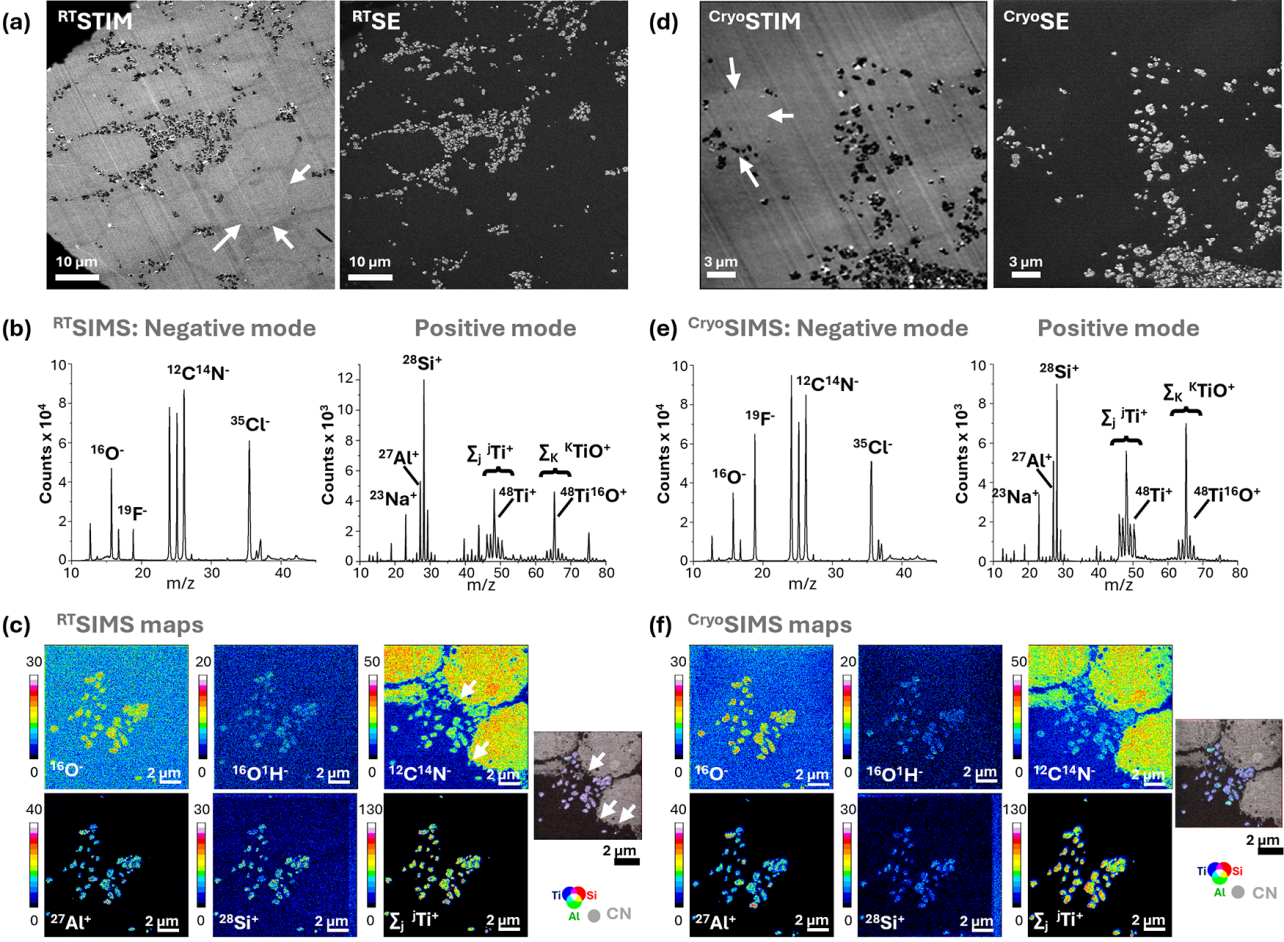
Correlative microscopy and microanalysis of resin-embedded cross sections of KeratinoSens cells exposed to 100 μg/mL AlSiTiO_2_ nanoparticles analyzed at RT and under cryogenic conditions (cryo; <−140 °C). (a, d) While He-based SE imaging allows identification only of the nanoparticles protruding from the flat sections, He-based STIM imaging obtained at the same ROI allows identification of biological structures by mass contrast (exemplary cells highlighted by white arrows), even in unstained samples. In elemental SIMS, obtained at a different area and using the Ne beam (b, e), biological structures rich in proteins are highlighted using the CN^–^ cluster signal at *m*/*z* = 26 available in negative polarity SIMS. The SiAlTiO_2_ nanoparticles are visualized by all three represented elemental signals (*m*/*z* = 27 for Al^+^, 28 for Si^+^ and 46–50 for Ti^+^ and *m*/*z* = 62–67 for TiO^+^, respectively) in positive polarity SIMS. (c, f) SIMS mapping shows that most particles are only touching the surface of the cells where they are in direct contact with the microvillar cell protrusions (some marked by arrows in the ^12^C^14^N map). Maps are displayed in linear scale. The intensity bar corresponds to the ion signal intensity in counts.

While SE imaging showed predominantly particulate information on such flat sections, STIM analysis offers additional insights based on mass contrast (white arrows in Figures 4a and d for RT and cryo-analysis, respectively). Note that biocellular structures were visible in these samples even without the addition of on-section heavy metal contrast enhancements, which are typically used for biological TEM preparations.

Thus, areas showing cell-particle interaction were easily determined and selected for SIMS analysis (SIMS mass spectra Figure 4b and e, and corresponding mass-filtered maps in Figure 4c and f, for RT and cryo-conditions, respectively).

Both RT-SIMS and cryo-SIMS revealed significant peaks attributed to, e.g., ^16^O^–^ and ^12^C^14^N^–^ for general biological contrast in negative mode. In positive mode, the most prominent peaks corresponded to ^27^Al^+^, ^28^Si^+^, ^48^Ti^+^ and ^48^Ti^16^O^+^ and are representative of the nanoparticle composition. While most SiAlTiO_2_ particles are accumulating in extracellular space, occasionally in proximity to cell protrusions (See arrows in the ^12^C^14^N map of Figure 4c), some particles were localized inside cells, suggesting endocytic activity (e.g., arrows in Figure 4d, show a cell with internalized particles, see also Supplementary Figure S7).

These investigations confirm the stability of the acquisition conditions independent of the temperature of the sample. A slightly blurred image in the SIMS maps under cryo-conditions suggests a minor amount of recondensation of water and carbon on the sample surface. However, potentially increased ^16^O^–^ and ^17^OH^–^ signals that could point to ice formation during cooling experiments with respect to RT investigation, were not observed. As SIMS is *per se* a destructive technique and as quantitative analysis is not readily available, a deeper analysis of smaller changes in the mass ratios within the mass spectra obtained consecutively in the same ROI was not performed at this stage. Notably, the cryo-SIMS signal appears to be slightly decreased, especially in positive mode, in comparison to RT. This could be attributed to different mechanisms, such as a change in local sample surface charging state caused by a change in sample conductivity, impacting the local surface potential and extraction field. Changes in sample atom ionization processes and related ionization efficiencies can also play a role at these considerably lower temperatures with respect to RT. In addition, the sample sputtering process and resulting sputtering yields are impacted due to changes in interatomic bonding strengths. It is known from literature that the sputtering yield for several materials is reduced in the temperature range of −130 °C to −200 °C in comparison to RT, e.g., low melting point metals,^39^ group III and V semiconductors^40^ and polymers^41^ (to which the resin embedded sample investigated here is most comparable). Overall, qualitative assessment indicates that similar data can be obtained from samples investigated at RT or in cryo-conditions. More detailed experimental as well as theoretical studies would be needed to draw more concise conclusions about these phenomena, but are beyond the scope of this work.

### Investigation of Frozen-Hydrated Lamella under Cryogenic Near-to-Native Conditions

Chemical analysis of resin-embedded biological specimens can be used to study exposure to nanoparticles on the cellular level. This is important to assess, for example, interactions with potentially harmful particles such as microplastics or asbestos. However, the investigation of metabolic changes or the exposure of cells to soluble substances (drugs, or other small molecules) is problematic in resin-embedded specimens, since they can be washed away during sample preparation. Therefore, chemical analysis of vitrified cells provides a new avenue to *in situ* analytical cell biology and biomedicine.

As proof of suitability to perform elemental analyses on frozen-hydrated specimens in the npSCOPE, cell cultures were first grown directly on TEM grids, plunge-frozen, and thin cryo-lamellae were prepared by cryo-FIB milling with a Ga^+^ beam before transferring the samples into npSCOPE. SE imaging (Figure 5a,b) was used to locate cryo-lamellae for cryo-SIMS mapping (Figure 5c). Ice crystals were occasionally observed on the surface of the lamellae. These crystals originated from contact with LN_2_ during sample storage and transfer between Aquilos 2 and the npSCOPE. Moisture deposition on the sample surface is presumably negligible considering the high vacuum in the chamber, the shield acting as a cold trap, and the on-grid contamination results presented in the supplements (see Supplementary Figure S2 and Supplementary Table S1). However, redeposition of hydrocarbons remains possible. Although direct correlation to TEM data sets was not performed in this work, the different isotopic maps retrieved show significant contrast for subcellular details. While ^16^O^–^ and ^16^O^1^H^–^ give significant signal predominantly on smaller ice crystals residing on the surface of the sample (black-outlined triangles in Figure 5; larger crystals lack signal due to charging, white arrows), O^–^ has also an additional unique contrast in the lower right corner of the lamella where it overlaps with the ^12^C^14^N^–^ signal suggesting biological contrast. On the other hand, some vesicle-like features are especially highlighted by CN^–^ (white-outlined triangles).

**Figure 5:**
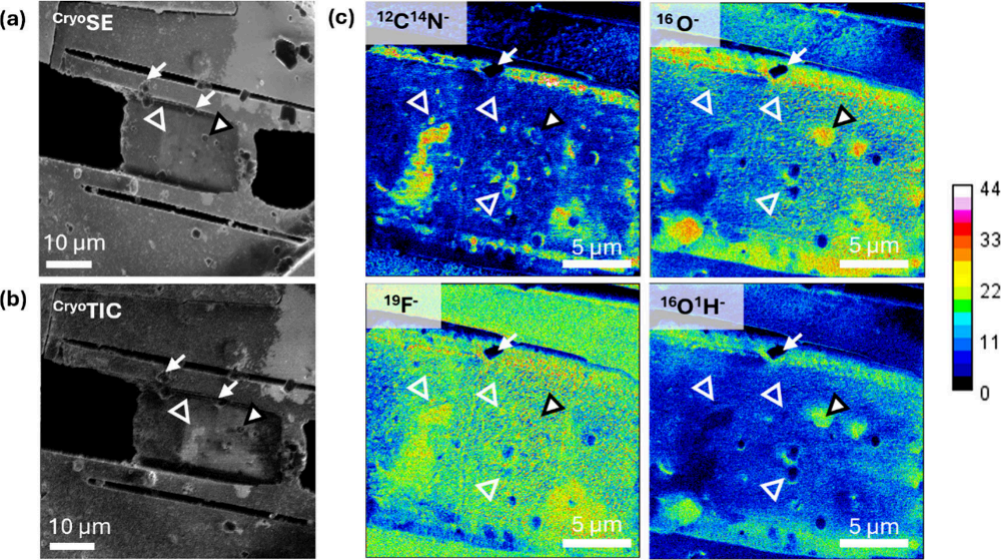
Correlative cryogenic SE-SIMS of frozen-hydrated lamella of A549 cells (a) He-based SE image of the whole cryo-lamella (b) Total ion count detector (TIC) signal in negative polarity SIMS operation obtained with the Ne beam, and (c) exemplary SIMS maps, showing biological contrast at *m*/*z* = 16, 17, 19, and 26. Small arrows and black-outlined triangles point to larger or smaller ice crystals, respectively (the latter being rich in O^–^, OH^–^), which formed during sample preparation and transfer, on top of the sample surface. White-outlined triangles point to several vesicular structures, which are high in CN^–^.

The data demonstrates the general suitability of Neon beam-based FIB-SIMS imaging for biological applications and provides a foundation for future investigations including studies on metallic nanoparticle uptake in nanotoxicological research settings at the highest possible spatial resolution. However, if molecular information is needed for drug interaction-based or metabolic studies, isotopic labels need to be introduced into the sample for elemental SIMS. If sub-100 nm spatial resolution is not needed for the analysis, molecular mass spectrometric approaches with higher selectivity and mass resolution are available and more suited (covering typically hundreds of nanometer to micrometer spatial resolution, e.g. MALDI, TOF- or ORBI-SIMS). The 3D TOF/OrbiSIMS has recently bridged the gap in the resolution scale between NanoSIMS and MALDI^42^ and is capable of analyzing frozen-hydrated samples.^43^

## Conclusion

High-resolution scanning He^+^ and Ne^+^ ion imaging and analysis at cryogenic temperatures, as it is described here, offers reduced beam damage for certain categories of RT-stable materials as compared to the respective analyses at RT. The novel cryo-stage/cryo-load lock installation exhibits extended temperature stability and negligible ice growth on cold samples even for multiday experiments. Hence, this new setup paves the way for multimodal analyses with improved structural preservation for in-depth chemical analysis of nanoparticulate and beam-sensitive materials, as well as frozen-hydrated biological specimens. Importantly, the npSCOPE platform allows topographic, bulk and isotopic elemental surface information (including Li and H signals) to be obtained at the highest possible spatial resolution for SIMS, all within the same instrument and under cryogenic conditions. This is important for a number of materials that can only be studied at lowered temperatures due to phase transitions, such as solid–liquid interfaces in batteries,^44^ or phases that only exist at cryogenic temperatures.^45^ Reducing diffusion in samples is also valuable to either study chemical distributions for highly diffusible elements or reduce the influx of undesired external elements (such as H).^46^ Finally, the reduction of the working temperature can significantly reduce thermal scattering of the ion beam, allowing crystalline structures to be studied more effectively.

Cryo-SIMS analysis on cryo-lamellae of biological specimens provides elemental mapping of subcellular features that can in the future be correlated with any prior cryo-ET experiments. While molecular mass spectrometry at cryogenic temperature allows for more detailed structural information on chemical composition and metabolic changes of the biological specimen, only elemental SIMS as employed here, achieves the probe sizes and respective spatial resolution and sensitivity necessary to identify even single metallic nanoparticles or structures smaller than 50 nm, which to our knowledge, are not yet accessible by molecular MS. STIM analysis at varying temperatures allows for not only beneficial ion channeling analyses for fundamental studies in materials science, but also offers additional capabilities toward dark field imaging and time-of-flight experiments, which are currently being explored.

For the first time, a fully integrated workflow for correlative ultrastructural imaging and mass spectrometric analysis at the highest possible spatial resolution within one instrument is presented. Now, the whole world of frozen-hydrated or gel-like specimens is in reach, suggesting near-to-native nanotoxicological and biomedical analyses without redistribution or washout of elements. Future developments will push the limits even further toward higher mass resolution and reduced sample fragmentation crucial for full subcellular molecular insight.

## Supplementary Material



## Data Availability

Upon publication, the data used in this paper are available at doi.org/10.5281/zenodo.17252193

## Notes

Additional experimental details include (1) detailed methods regarding sample transfer under oxygen and moisture-free atmosphere, (2) more detailed method description and respective results showcasing the lack of ice contamination over time, (3) a more detailed method description for correlative analysis of samples by SE, STIM and SIMS at RT and under cryo-conditions, (4) additional examples for beam damage in Silica-coated Au nanoparticles and the related quantitative study of line profiles, and (5) additional images depicting the intracellular uptake of SiAlTiO_2_ particles. (PDF)
