# Supplementary material for: Cryo-HIM-SIMS on the npSCOPE: Correlative Topographic, Transmitted and SIMS Imaging at Cryogenic Temperatures
Source: Anal Chem. 2026 Apr 20;98(17):12317–27. doi: 10.1021/acs.analchem.5c06908 (PMC13150799; doi:10.1021/acs.analchem.5c06908)
Supplement: Supplementary file 1 [file ac5c06908_si_001.pdf]

Supporting Information (SI)

Cryo-HIM-SIMS on the npSCOPE: Correlative topographic, transmitted and SIMS imaging at cryogenic temperatures

Tatjana Taubitz[1, †], Olivier De Castro[1], Dustin Andersen[1], Zahraa Berro[1,2], Sukriti Hans[1], Saba Tabean[1], Moritz Wachsmuth-Melm[3,4], Gerhard Hobler[5], Inge Nelissen[6], Falk Lucas[7], Santhana Eswara[1], Petr Chlanda[3,4], Tom Wirtz[1] Jean-Nicolas Audinot and Antje Biesemeier[1]*

[1] Scientific Instrumentation and Process Technology, Advanced Instrumentation for Nano-Analytics (AINA), Luxembourg Institute of Science and Technology, 4422 Belvaux, Luxembourg

[†] Department of Structural Biochemistry, Max Planck Institute of Molecular Physiology, 44227 Dortmund, Germany.

[*] Corresponding author: antje.biesemeier@list.lu; Scientific Instrumentation and Process Technology, Advanced Instrumentation for Nano-Analytics (AINA), Luxembourg Institute of Science and Technology, 4422 Belvaux, Luxembourg

[2] Doctoral Program in Systems and Molecular Biomedicine of the DSSE, University of Luxembourg, 4365 Belval Esch-sur-Alzette, Luxembourg

[3] Department of Infectious Diseases-Virology, Medical Faculty, Heidelberg University, 69120 Heidelberg, Germany

[4] BioQuant - Center for Quantitative Analysis of Molecular and Cellular Biosystems, Heidelberg University, 69120, Heidelberg, Germany

[5] Institute of Solid-State Electronics, Technische Universität Wien, 1040 Wien, Austria

[6] Environmental Intelligence, Vlaamse Instelling voor Technologisch Onderzoek (VITO), 2400 Mol, Belgium

[7]ScopeM, ETH Zürich, 8093 Zürich, Switzerland

Contents

Supporting information ....**Error! Bookmark not defined.**

Supplementary Method 1: Sample transfer under oxygen and moisture-free atmosphere – Cryo-glovebox and cryo-transfer suitcase....................................... 3

Supplementary Method 2 and respective results on the lack of ice contamination over time 5

Supplementary Method 3: Correlative analysis of samples by SE, STIM and SIMS at RT and under cryo-conditions 7

Supplementary Results 1: Results and quantitative analysis of beam induced alteration of Silica-coated Au nanoparticles: 9

Supplementary Results 2: Intracellular uptake of $SiAlTiO_2$ particles 16

Supplementary Method 1: Sample transfer under oxygen and moisture-free atmosphere – Cryo-glovebox and cryo-transfer suitcase

A common source of artefacts in cryo-microscopy is the formation of ice crystals on the sample, mostly occurring due to ambient humidity during sample transfer. Therefore, a sample transfer system was developed that minimizes the risk of contaminant ice formation by reducing the humidity as much as possible. The cryo-glovebox prototype used here achieves a dry nitrogen atmosphere (below 1 ppm H_2O) and utilizes a UHV cryo-transfer suitcase that allows sample transfer at temperatures < -190° C and vacuum in the 10^{-10} mbar range (supplementary figure 1). The cryo-glovebox has two insertion chambers to introduce tools and samples and is equipped with an LN_2 basin for sample manipulation (e.g., loading the sample grids onto the sample holder) and an external pedal to control the fill level in the basin. The basin is displaceable and can either be positioned close to the gloves to allow for easy sample handling or can be placed underneath the cryo-loading station for sample transfer into the cryo-transfer suitcase. The cryo-loading station is equipped with an internal cryo-shield (T < -170° C) that is automatically cooled by LN_2 via an attached cold finger coming from an installed dewar. An integrated electric sample lift brings the sample holder up from the LN_2 basin into the cryo-loading dock. Once the cryo-loading station is evacuated to high vacuum/ultra-high vacuum level, the sample holder can then be transferred to the UHV cryo-transfer suitcase. The latter is equipped with a combined non-evaporable getter and ion pump driven by a battery-powered controller, and an LN_2 dewar attached to a dedicated cryo-shield comprising a direct contact sample cooling mechanism. Thereby, the sample can be kept under cold UHV conditions for longer periods without risking contamination. The UHV cryo-transfer suitcase can then be detached from the docking station and mounted to the cryo-load lock on the npSCOPE and into the cryo-stage of the npSCOPE.

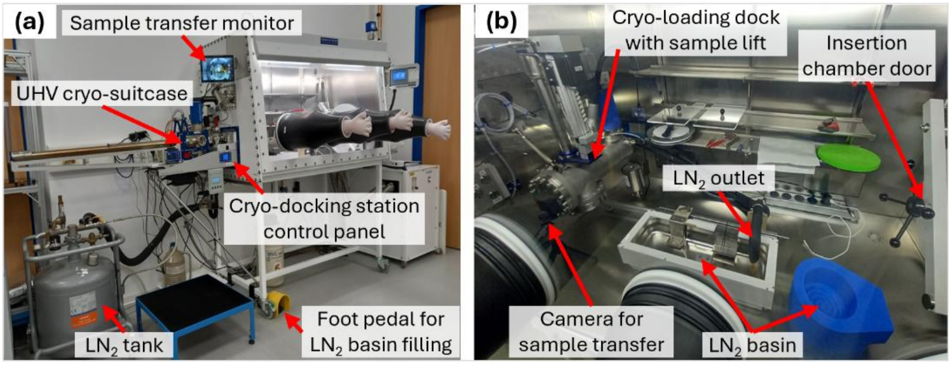

Figure S1: Cryo-glovebox prototype. (a) Cryo-glovebox with the cryo-transfer suitcase attached to the docking station. (b) Inside of the cryo-glovebox showing the cryo-loading dock and the LNs basin for sample manipulation and grid loading.

Supplementary Method 2 and respective results on the lack of ice contamination over time

Method: An empty 200 mesh holey carbon TEM Cu grid (PLANO, Wetzlar, Germany) was introduced into the npSCOPE under RT conditions. The instrumental setup was then cooled down and the grid inspected with SE imaging at 20 kV acceleration voltage of the He^{+} beam at a tilt of 20°. Potential ice buildup was assessed at various magnifications and after different time points during cool down and for another 2 days at cryogenic temperature. Dimensional measurements were done using Fiji/ImageJ. The estimated measurement error is ±0.005 µm, based on image pixel resolution and calibration.

Result: The npSCOPE chamber operates in the 10^{-7} Torr range and reaching 10^{-8} Torr under cryogenic conditions (i.e. < -139°C in this work). Thus, humidity and corresponding ice formation during cryogenic operation can be minimized. In addition, the integrated cryo-shield (-187°C) works as a cold trap as it is always kept colder than the cryo-stage. To validate the quality of the low humidity vacuum setup, an empty TEM grid was introduced at RT and then cooled down and monitored over three days at cryogenic conditions. Frequent SE imaging (supplementary figure 2) was used to monitor changes in grid topography due to potential ice build-up at different magnifications over time. Red double arrows in the figure depict four locations where distance measurements were obtained for quantitative assessment (see also supplementary table 1). Any increase of these distances would suggest significant ice formation (even if not visible by purely topographic observation). However, the data confirms stable low humidity conditions without significant ice contamination over time. This consistency is crucial for reliable, high-resolution imaging of sensitive samples, including their topographic aspects and ensuring chemical integrity of the sample throughout extended cryogenic experiments.

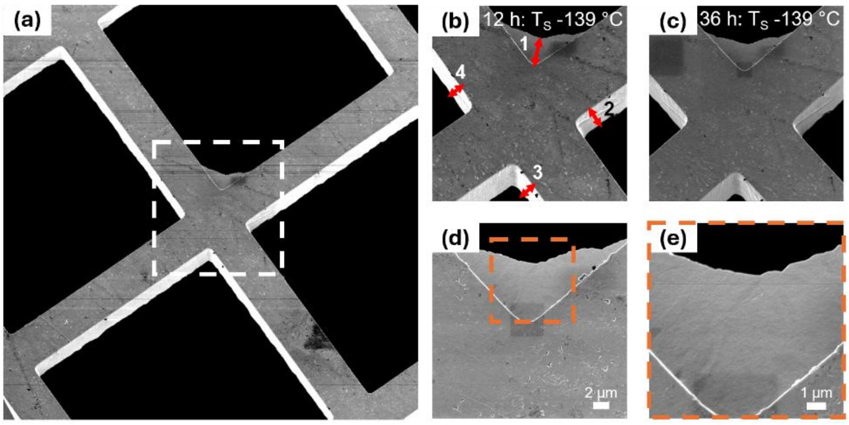

Figure S2: Monitoring ice build-up over time: Consecutive SE images of a TEM grid at 20° tilt. (a) Overview at room temperature (RT). (b) 12h at –139 °C stage temperature, (c) 36 hours at –139 °C. (d) Further magnified SE image of a section from (c). (e) Final zoom of a section from (d) with FOV 10 µm, highlighting the complete absence of ice buildup at the edges. Red double errors depict location of distance measurements for supplementary Table 1.

Table S1: Distance measurements (in nm) at four fixed locations (as marked in supplementary figure 2 (b)) over time. Shield temperature at constant -187 °C, stage temperature ≤ -139 °C throughout the experiment, stage tilt constant at 20°.

	**Distance at location (nm)**			
**Time Point**	1	2	3	4
**12 h**	2.907	1.854	1.625	1.365
**18 h 39**	2.917	1.812	1.656	1.375
**36 h**	2.902	1.849	1.642	1.369

Supplementary Method 3: Correlative analysis of samples by SE, STIM and SIMS at RT and under cryo-conditions

In short and as discussed in De Castro. *et al.* npSCOPE: A New Multimodal Instrument for In Situ Correlative Analysis of Nanoparticles. *Anal Chem* **93**, 14417–14424 (2021), correlative SE, STIM and SIMS analysis can be performed on the npSCOPE thanks to the GFIS imaging and analytics capabilities. While high-resolution SE imaging with He^+ benefits from a short working distance between the sample and the ion column (here 7.6 mm), SIMS analyses with the Ne^+ beam require the working distance to be increased to 18 mm (to accommodate the ion-extraction optics) and a sample bias of +/- 500 V to be applied. STIM can be performed also using the finely-focused He^+ beam settings for optimal spatial resolution on sufficiently thin samples (typically less than 100 nm thick for 30 keV He^+). In STIM samples with holes, the beam current has to be lowered to less than 0.1 pA to avoid MCP/DLD detector overload. Supplementary table 2 describes typical acquisition conditions used throughout all experiments at RT and under cryogenic conditions. All samples were investigated using these standard conditions if not described otherwise in the respective sections. Images were treated in Image J using *brightness/contrast*. For STIM imaging of delicate biological samples, a lower signal-to-noise ratio was compensated by collecting multiple images (2-3) and then combining them with Fiji/ImageJ and applying a light Gauss blur (*Images to stack,* followed by *Z project*; *Gauss blur filter* set to 0.5 pixels).

Table S2: Typical acquisition conditions for SE, STIM and SIMS analysis. Acquisition conditions do not need to change at different temperatures for beam sensitive samples. For frozen-hydrated specimens, dwell times might need to be reduced in case of melting depending on the individual sample thicknesses.

	^{He}SE	^{He}SE+ SIMS box	^{Ne}SIMS neg	^{Ne}SIMS pos	^{He}STIM
**Acceleration (keV)**	20 - 30	20 - 30	20	20	30
**Gun pressure (mbar)**	$2.6x10^{-6}$	$2.6x10^{-6}$	$2.6x10^{-6}$	$2.6x10^{-6}$	$2.6x10^{-6}$ - $2.6x10^{-7}$*
**Beam current (pA)**	0.5 - 5	0.5 - 5	< 10	< 10	0.1 – 0.5
**Working distance (mm)**	7.6	18	18	18	7.6 / 18
**Dwell time (µs)**	2	2	8000	3000	200
**Frame averaging**	8	8	-	-	1 - 4
**Image size (pixel x pixel)**	512	512	512	512	512

* For STIM on sample containing holes, like holey carbon film, the beam current is to be reduced to less than 0.1 pA to avoid beam damage on the MCP/DLD detector surface and detector overloading. This is done by lowering the gun pressure, as here also the contribution of neutrals to the STIM signal is decreased.

Supplementary Results 1: Results and quantitative analysis of beam induced alteration of Silica-coated Au nanoparticles:

To perform a quantitative analysis of the induced particle alterations under room temperature and cryo-conditions imaging, two parameters have been studied in more detail. Namely, the proportional change of intensity valley dip at neighboring particle borders from image frame 1 in comparison to frame 10. As well as the proportional change in interparticle gap. Both parameters are illustrated schematically in supplementary figure 3. The considered intensity valley dip has been calculated as average value for each particle border, as in most cases the particles edge intensity maximum Imax differed from one side of the border to the other. Additionally, the dip has been expressed as ratio (in percent) of the corresponding particle edge intensity (left or right edge), before the average has been calculated. The proportional change of this average $\overline{\Delta_{\%}}$ has then been studied from frame 1 to frame 10. The gap between two particles G at the respective border is defined as distance between both positions of the respective particle edge intensity maxima. The proportional change of this gap value then has also been studied when going from frame 1 to frame 10.

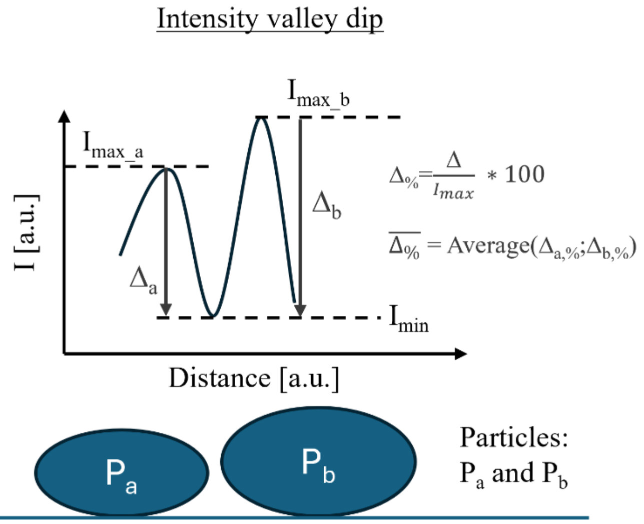

(a)

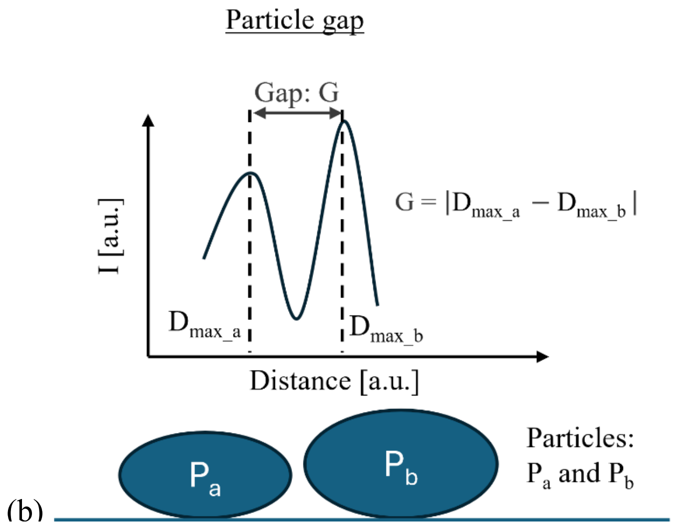

Figure S3. Schematic representation of the parameters used for the quantitative investigation of He-beam induced particle changes/modification under SE repeated imaging: (a) Intensity valley dip at particle border; (b) Particle gap at border.

More details of this quantitative parameter variation study can be found here below in supplementary table 3. A graphical representation of the results is shown additionally in supplementary figure 4.

Table S3: Quantitative parameter variation study for inspection of He ion beam-induced particle alteration under room and cryo-temperature SE imaging.

Room temperature imaging								
Set	Border	Frame	$\Delta_{a,\%}$ [%]	$\Delta_{b,\%}$ [%]	$\overline{\Delta_{\%}}$ [%]	Proportional change of $\overline{\Delta_{\%}}$ (frame10/frame 1) [%]	Gap [pixels]	Proportional change of gap (frame10/frame1) [%]
1	P1 – P2	1	18.24	22.99	20.62	/	17.5	/
1	P1 – P2	10	1.16	9.79	5.48	-73.44	10.5	-40.00
1	P2 – P3	1	18.28	14.88	16.58	/	18.5	/
1	P2 – P3	10	2.85	9.38	6.12	-63.09	13.5	-27.03
1	P3 – P4	1	13.76	18.79	16.28	/	20	/
1	P3 – P4	10	-19.75	14.16	-2.80	-117.18	12	-40.00

2	P1 – P2	1	7.94	23.87	15.90	/	14.5	/
2	P1 – P2	10	-11.95	13.44	0.74	-95.33	16	10.34
2	P2 – P3	1	53.20	51.90	52.55	/	21	/
2	P2 – P3	10	49.07	51.53	50.30	-4.28	16	-23.81
3	P1 – P2	1	8.49	15.30	11.89	/	14	/
3	P1 – P2	10	-35.37	20.72	-7.33	-161.61	17.5	25.00
3	P2 – P3	1	8.63	5.24	6.94	/	10	/
3	P2 – P3	10	0.23	0.40	0.32	-95.43	3	-70.00
				Overall average		-87.19	Overall average	-23.64

Cryo-temperature imaging								
Set	Border	Frame	$\Delta_{a,\%}$ [%]	$\Delta_{b,\%}$ [%]	$\overline{\Delta_{\%}}$ [%]	Proportional change of $\overline{\Delta_{\%}}$ (frame10/frame1) [%]	Gap [pixels]	Proportional change of gap (frame10/frame1) [%]
1	P1 – P2	1	44.75	42.95	43.85		20	
1	P1 – P2	10	55.07	54.08	54.57	24.46	21	5.00
2	P1 – P2	1	8.04	6.67	7.35		12.5	
2	P1 – P2	10	13.98	8.57	11.28	53.31	14.5	16.00
2	P2 – P3	1	8.92	7.34	8.13		14.5	
2	P2 – P3	10	6.99	3.18	5.08	-37.49	11.5	-20.69
3	P1 – P2	1	12.51	23.75	18.13		13	
3	P1 – P2	10	11.75	20.24	16.00	-11.76	17	30.77
3	P2 – P3	1	15.53	33.44	24.49		15.5	
3	P2 – P3	10	7.32	27.90	17.61	-28.07	19.5	25.81
3	P3 – P4	1	25.46	21.68	23.57		19	
3	P3 – P4	10	23.33	32.77	28.05	19.01	24.5	28.95
				Overall average		7.13	Overall average	7.77

Under room temperature conditions (for reference also see supplementary figure 4(a)), at all analysed particle borders, the proportional change of $\overline{\Delta_{\%}}$ is negative and reaches as an overall average a value of about -87%. Meaning that the valley dip in between particles decreases, pointing out that particle coalescence takes place during repeated imaging (prolongated ion beam exposure) during these conditions. Note that in some cases the proportional change even exceeds the -100 % limit, indicating that there is no more a valley in between the respective particles but rather a continuous hill slope has built up between neighbouring particles after the exposure in frame 10. Additionally, the gap G between particles is decreasing at the majority of the analysed borders and reaches an overall average in proportional change of about -24%.

On the other hand, at cryogenic conditions (for reference also see supplementary figure 4(b)), the proportional change of $\overline{\Delta_{\%}}$ reaches in average a slightly positive value of about 7%, indicating a general increase or rather stable situation in valley dip. Note that in none of the cases the proportional change of $\overline{\Delta_{\%}}$ comes near the valley to hill limit at -100% and rather varies about the 0% value, in comparison to the room temperature investigation where all values are in the negative range with the majority < -50%. Additionally, under cryogenic conditions the gap G between particles proportionally changes on average by about 8%, and at the majority of the investigated borders the proportional change is positive. Meaning that globally the gaps between particles open up, mainly due to the ion beam sputtering process, with the counteracting process of material fusion (particle coalescence) being more inhibited at these low temperatures in comparison to room temperature.

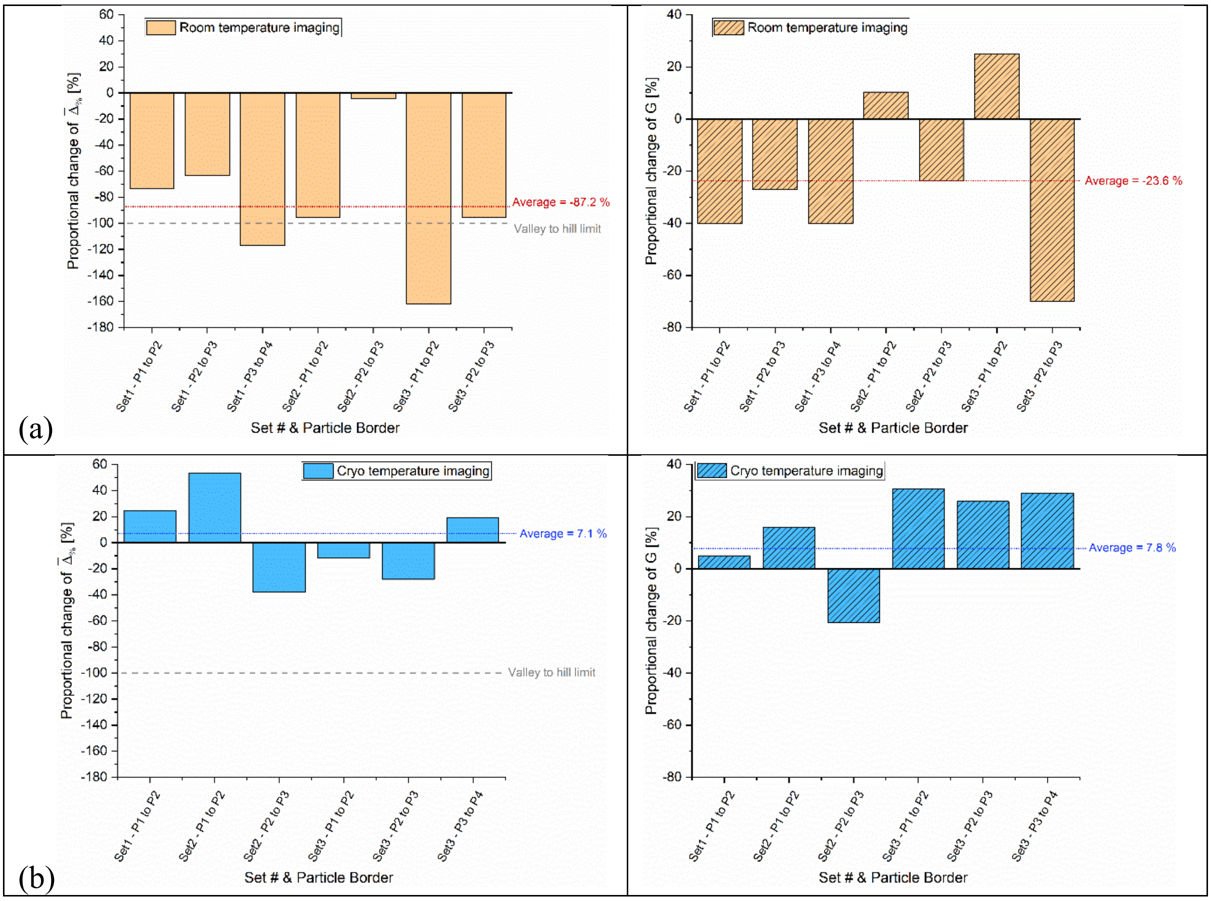

Figure S4. Graphical representation of results for proportional changes of the intensity valley dip average $\overline{\Delta_{\%}}$ and particle gap G parameters, occurring during He-based SE imaging series (frame 1 to frame 10) of Silica-coated Au nanoparticles at: (a) Room temperature; (b) Cryo-temperature.

Supplementary figure 5 and 6 are meant to illustrate all considered line scans and locations of the exact particle borders that have been taken into account for the performed quantitative analysis of the ion beam irradiation studies at room temperature and cryogenic temperature, respectively.

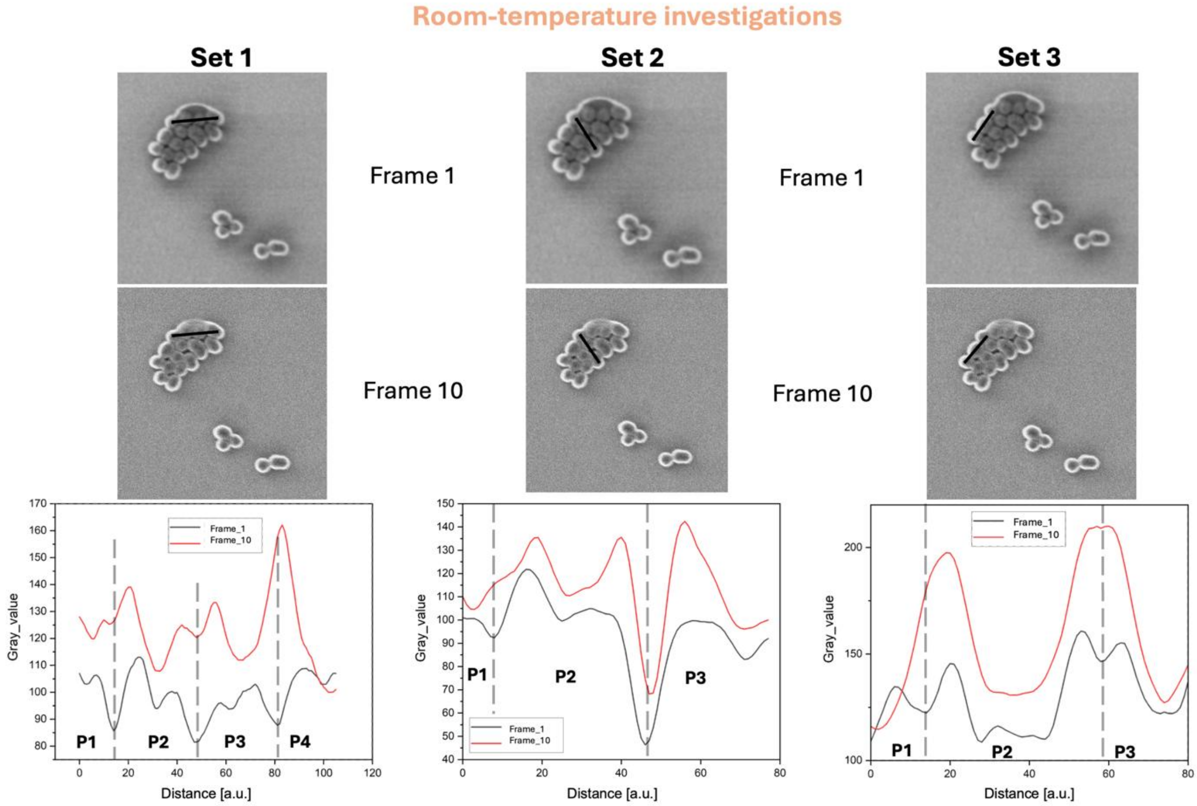

Figure S5. Additional line profiles extracted from a series of 10 SE images at different locations spanning over two or more particles showcasing He beam damage over time under RT conditions. 3 different regions (Set 1–3). Each set compares profiles from frames1 and 10. Image series obtained on 50 nm sized silica-coated gold nanoparticles: SE image series of 10 images, taken with He+, 30 kV, 0.7 pA, FoV (2×2) µm2, line average 8, dwell time = 2 µs, (1024×1024). Total ion dose 1.84x1016 ions/cm2.

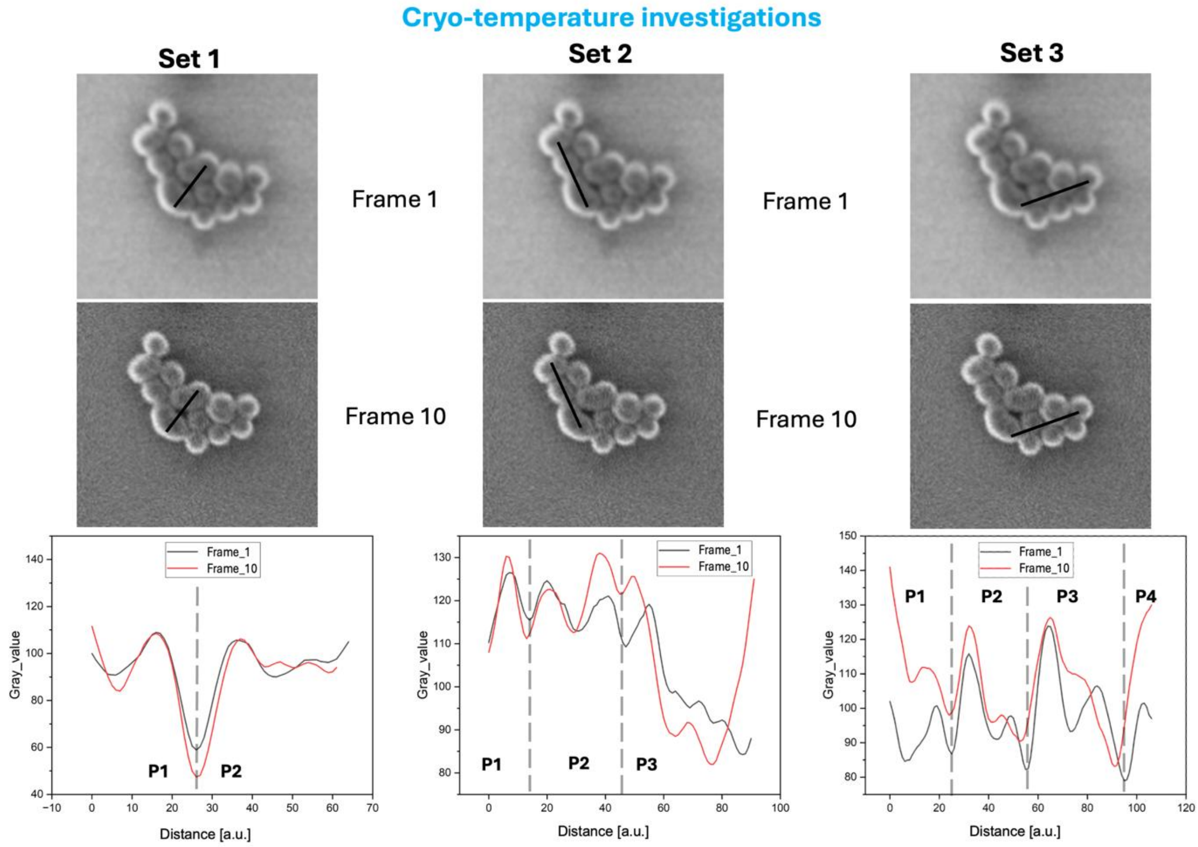

Figure S6: Additional line profiles extracted from a series of 10 SE images at different locations spanning over two or more particles acquired under cryogenic conditions showing improved stability/ little He beam induced damage under cryo-conditions. Three different regions (Set 1–3). Each set compares profiles from frames 1 and 10. Image series obtained on 50 nm sized silica-coated gold nanoparticles: SE image series of 10 images taken with He+, 30 kV, 0.7 pA, FoV (2×2) µm2, line average 8, dwell time = 2 µs, (1024×1024). Total ion dose 1.84x1016 ions/cm2.

Supplementary Results 2: Intracellular uptake of $SiAlTiO_2$ particles

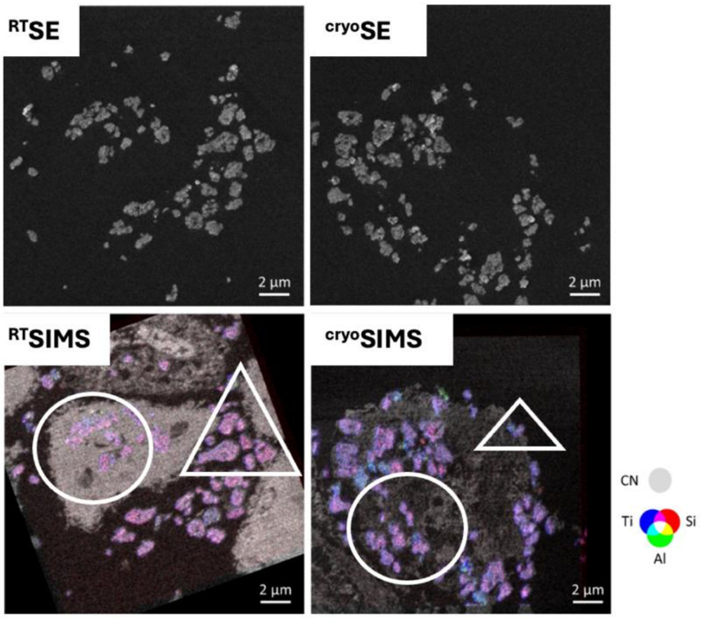

Figure S7: Additional examples of correlative microscopy and microanalysis of resin embedded cross sections of KeratinosensTM cells exposed to 100 µg/ml AlSiTiO2 nanoparticles at RT and under cryogenic conditions (cryo; <-140°C). While He+-based SE imaging (upper row) allows identification only of the nanoparticles protruding from the flat sections, Ne+-based SIMS (lower row) can visualize biological structures using the CN- cluster signal at m/z 26 available in negative polarity SIMS. The SiAlTiO2 nanoparticles are visualized by all three represented elemental signals (m/z 27 for Al, 28 for Si and 46-50 for Ti and m/z 62-67 for TiO2, respectively) in positive polarity SIMS. (c, f). SIMS mapping shows that most particles are only touching the surface of the cell cultures where they are in direct contact with the microvillar cell protrusions (triangles), while others are internalized (circles).
